# Supplementary material for: Abundant microchondrules in 162173 Ryugu suggest a turbulent origin for primitive asteroids
Source: Nat Commun. 2025 Jul 23;16:6466. doi: 10.1038/s41467-025-61357-1 (PMC12287358; doi:10.1038/s41467-025-61357-1)
Supplement: Supplementary file 1 — Supplementary Information [file 41467_2025_61357_MOESM1_ESM.pdf]

Supplementary Information

Abundant microchondrules in 162173 Ryugu suggest a turbulent origin for primitive asteroids

Matthew J Genge, Natasha V. Almeida, Matthias Van Ginneken, Lewis Pinault, Tobias Salge, Penelope J. Wozniakiewicz, Hajime Yano , Steven J. Desch

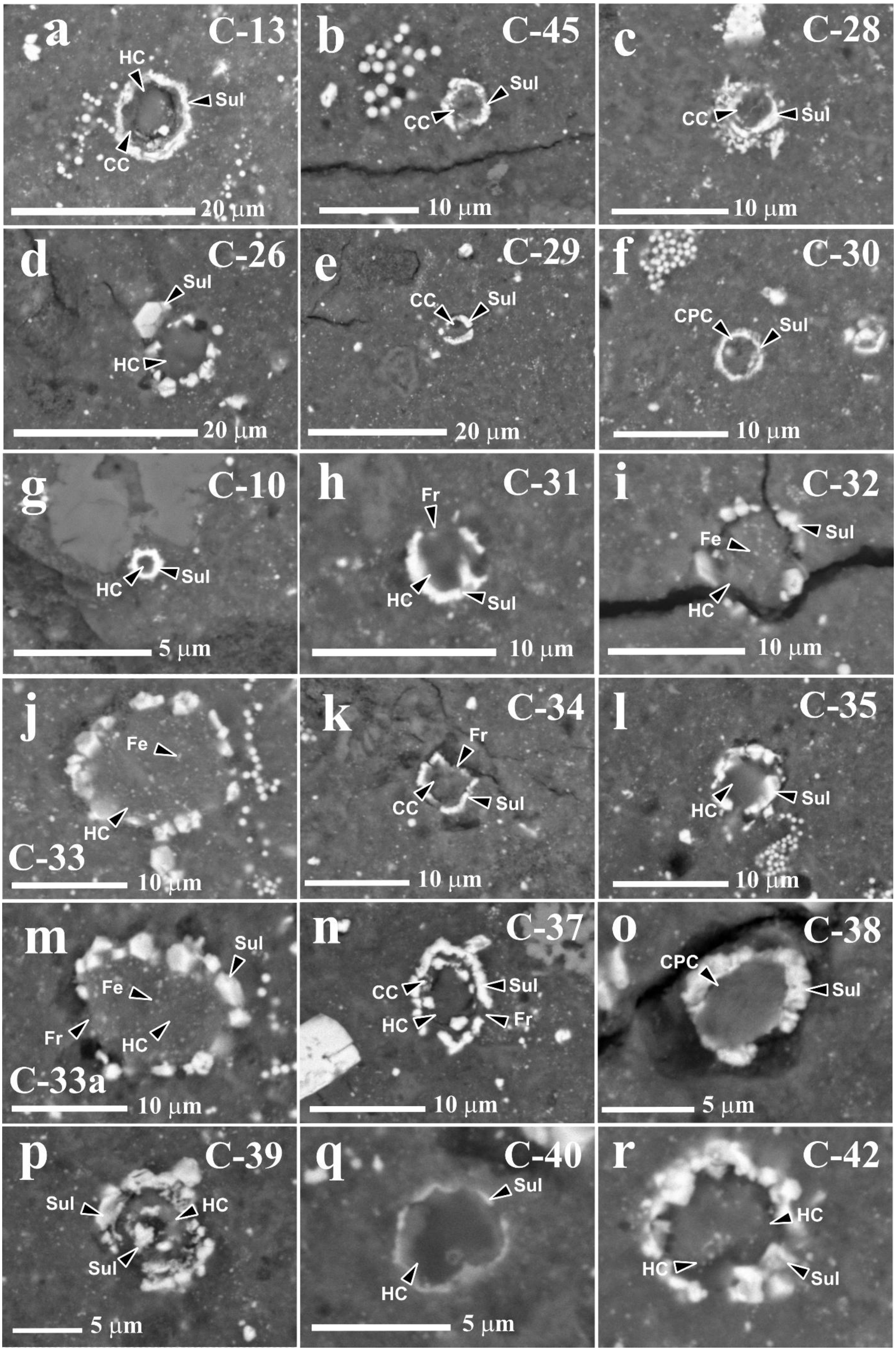
a C-13 HC Sul CC 20 μm
b C-45 Sul CC 10 μm
c C-28 CC Sul 10 μm
d C-26 Sul HC 20 μm
e C-29 CC Sul 20 μm
f C-30 CPC Sul 10 μm
g C-10 HC Sul 5 μm
h C-31 Fr HC Sul 10 μm
i C-32 Fe Sul HC 10 μm
j Fe HC C-33 10 μm
k C-34 Fr CC Sul 10 μm
l C-35 HC Sul 10 μm
m Sul Fe Fr HC C-33a 10 μm
n C-37 CC Sul HC Fr 10 μm
o C-38 CPC Sul 5 μm
p C-39 Sul HC Sul 5 μm
q C-40 Sul HC 5 μm
r C-42 HC HC Sul

Supplementary Figure 1. Backscattered electron images of sub-spherical objects in sample A0180. (a) C-13 has a homogeneous central silicate core surrounded by cryptocrystalline silicates with a sulphide rim. (b) C-45 has a polycrystalline sulphide-rim surrounding a core with a similar texture to the surrounding matrix except for the presence of more abundant sulphide. (c) C-28 has a discrete sulphide rim surrounded by dispersed sub-micron sulphide crystals. The core of the object is crypocrystalline with sheet-like crystal habits. (d) C-26 has a homogeneous ferromagnesian silicate core decorated by euhedral sulphides up to 1 µm in diameter and is similar to C-01 in Figure 1. (e) C-29 has a near complete rim of sulphide surrounding a cryptocrystalline silicate core. (f) C-30 has a complete rim of sulphide surrounding a core with sheet-like phyllosilicate with coarser crystal-size the surrounding matrix. (g) C-10 is a small object (1.5 µm in diameter) with a complete rim of sulphide exhibiting surface protrusions. The core of the object consists of homogeneous ferromagnesian silicates. (h) C-31 is a fractured SSO with a homogeneous core surrounded by a partial rim of sulphide. (i) C-32 is a rounded object consisting of a homogeneous core with sub-micron iron oxide inclusions decorated by micron-sized euhedral sulphide crystals. (j) C-33 is a relatively large (14 µm) object consisting of a homogeneous core with sub-micron iron oxides decorated by micron-sized sulphide crystsls. (k) C-34 is a fractured SSO with a thin (~0.5 µm) sulphide rim surrounding a homogeneous core. (l) C-35 has a facetted sulphide rim with prisms directed inwards (in contrast to C-33/C01) into a homogeneous silicate core. (m) C-33a is a second section through SSO C-33 exhibiting the change in shape of the object with depth into the specimen. It may be fractured along one surface. (n) C-37 has homogeneous core surrounded by a cryptocrystalline rim of sheet-like silicates and sulphides with an outermost 1 µm-thick rim of sulphide. The sulphide rim is disjointed in the lower part of the image suggesting insitu fracturing. (o) C-38 has an ellipsoidal core consisting of sheet-like phyllosilicates with crystal sizes larger than the surrounding matrix. The core is decorated by polycrystalline sulphide-rim. (p) C-39 has a homogeneous silicate core with sulphide inclusions surrounded by a double rim of sulphide. (q) C-40 has a homogeneous silicate core with an outer margin with a higher BSE potential surrounded by a 0.1 µm-thick rim of sulphide.(r) C-42 has a homogeneous silicate core with sub-micron iron oxide inclusions surrounded by a rim of sulphide crystals. Abbreviations: HC- homogeneous core, CC – cryptocrystalline core, CPC – coarse-phyllosilicate core, Fe – iron oxide, Sul – sulphide, Fr – fracture surface.

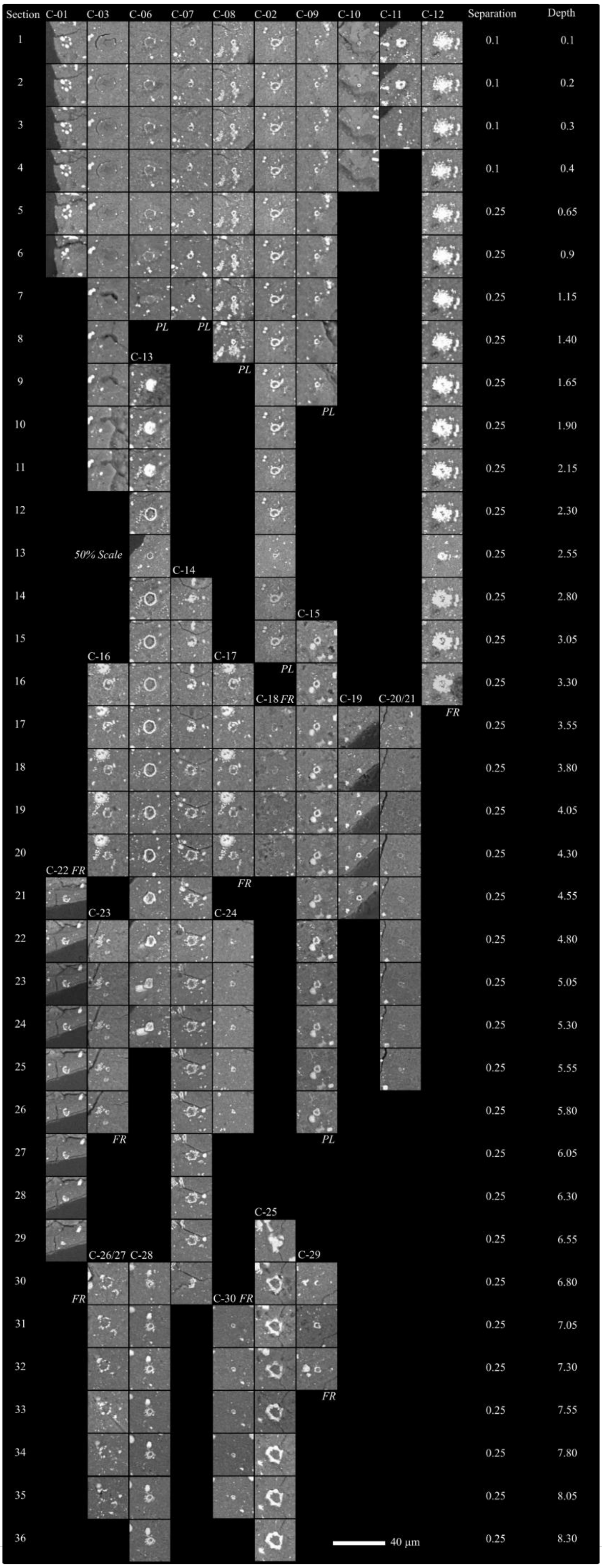
Section
C-01
C-03
C-06
C-07
C-08
C-02
C-09
C-10
C-11
C-12
Separation
Depth
PL
C-13
50% Scale
C-14
C-15
C-16
C-17
C-18 FR
C-19
C-20/21
FR
C-22 FR
C-23
C-24
C-25
C-26/27
C-28
C-29
C-30 FR
40 µm

Supplementary Figure 2. A stack of images of sub-spherical objects within sample A0180 obtained by serial sectioning. Twenty seven SSOs were observed within 35 planes of section with 2-10 SSOs observed in each section. Sub-spherical objects observed in each section are arranged horizontally whilst individual SSOs are aligned vertically. Objects are interpreted to have plucked out if a cavity is observed in the next section and are marked by PL. Where a cavity is not observed the SSO is interpreted to be fractured and is marked by FR. Depth estimates were obtained by calibrating to spheroidal magnetite and have ~30% errors. Separation of planes of section are based on polishing time. A constant image resolution is used except for section 13 which was images at 50% scale. All SSOs meet the criteria for microchondrules except C-25, which has a facetted outline.

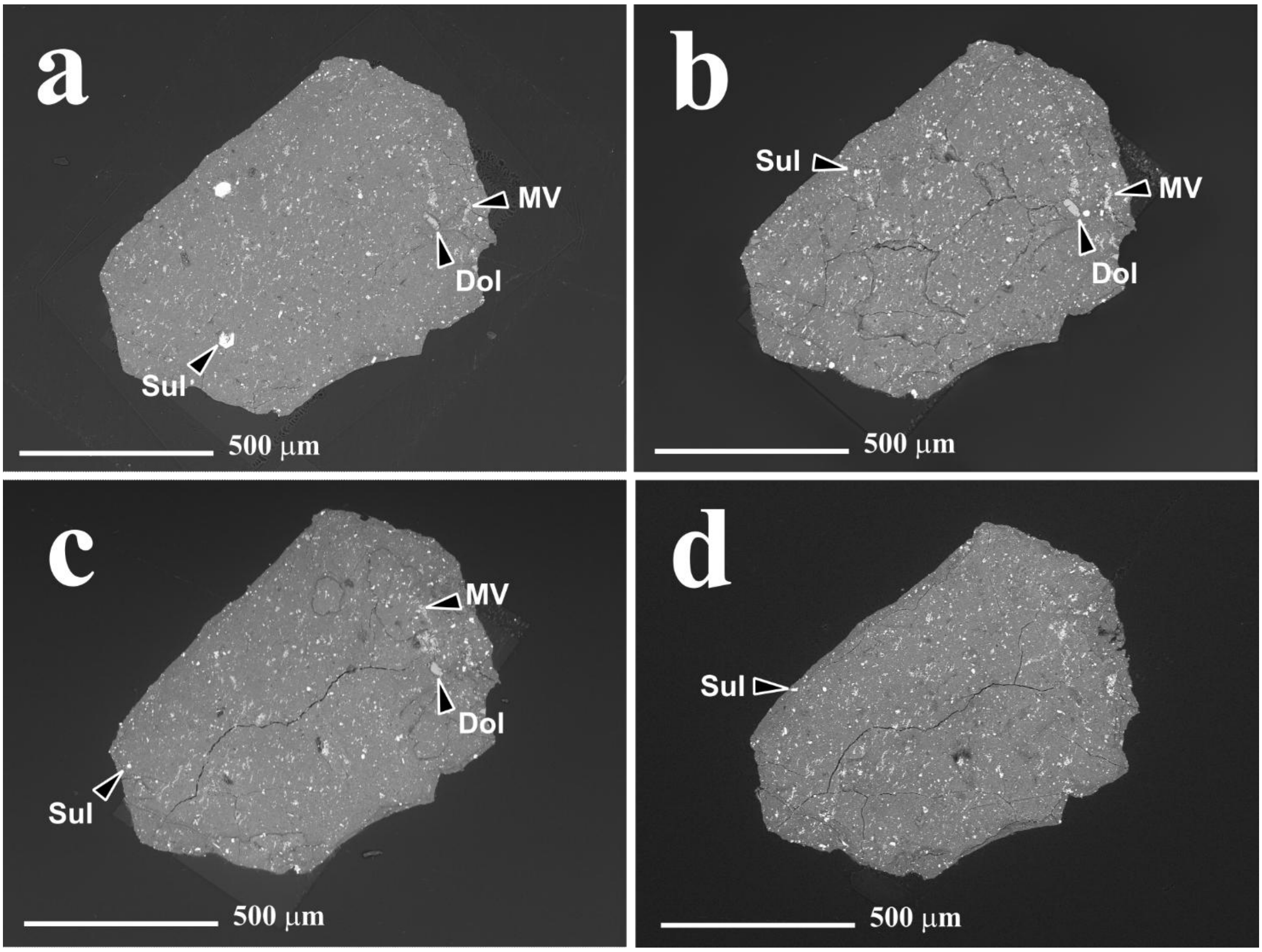

Supplementary Figure 3. A backscattered images of 4 polished sections of A0180. (a) Section 1, (b) Section 6 (6 µm depth), (c) Section 12 (2.3 µm depth), (d) Section 24 (5.05 µm depth). Depth's have uncertainties of ~30%. Abbreviations: Dol – dolomite, Sul – sulphide, MV – magnetite veins.

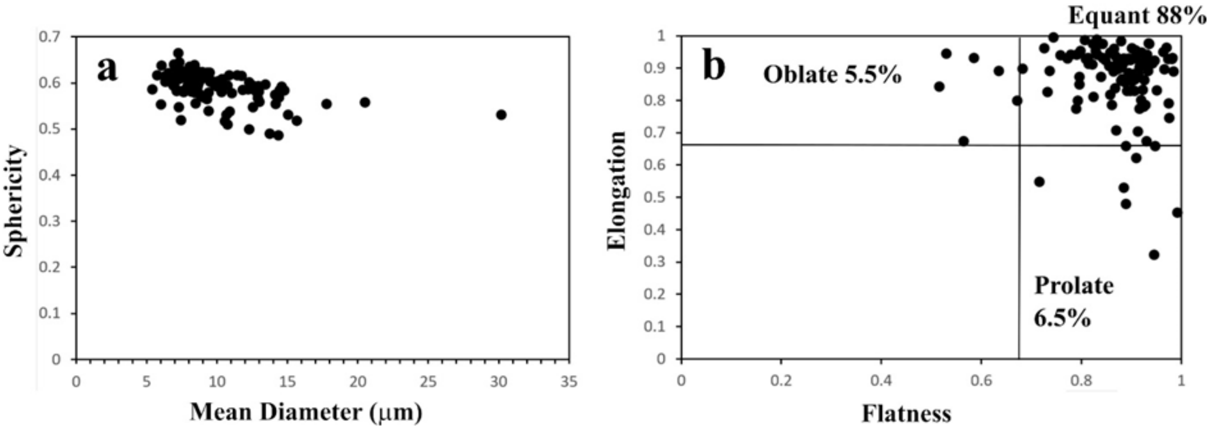

Supplementary Figure 4. Shape factors of microchondrules derived by measuring nano-XCT reconstructions. (a) Sphericity shows a decrease with increasing mean diameter consistent with surface tension controls on liquid droplets. (b) Elongation against flatness shows most microchondrules are equant with approximately the same abundance of oblate and prolate shapes.

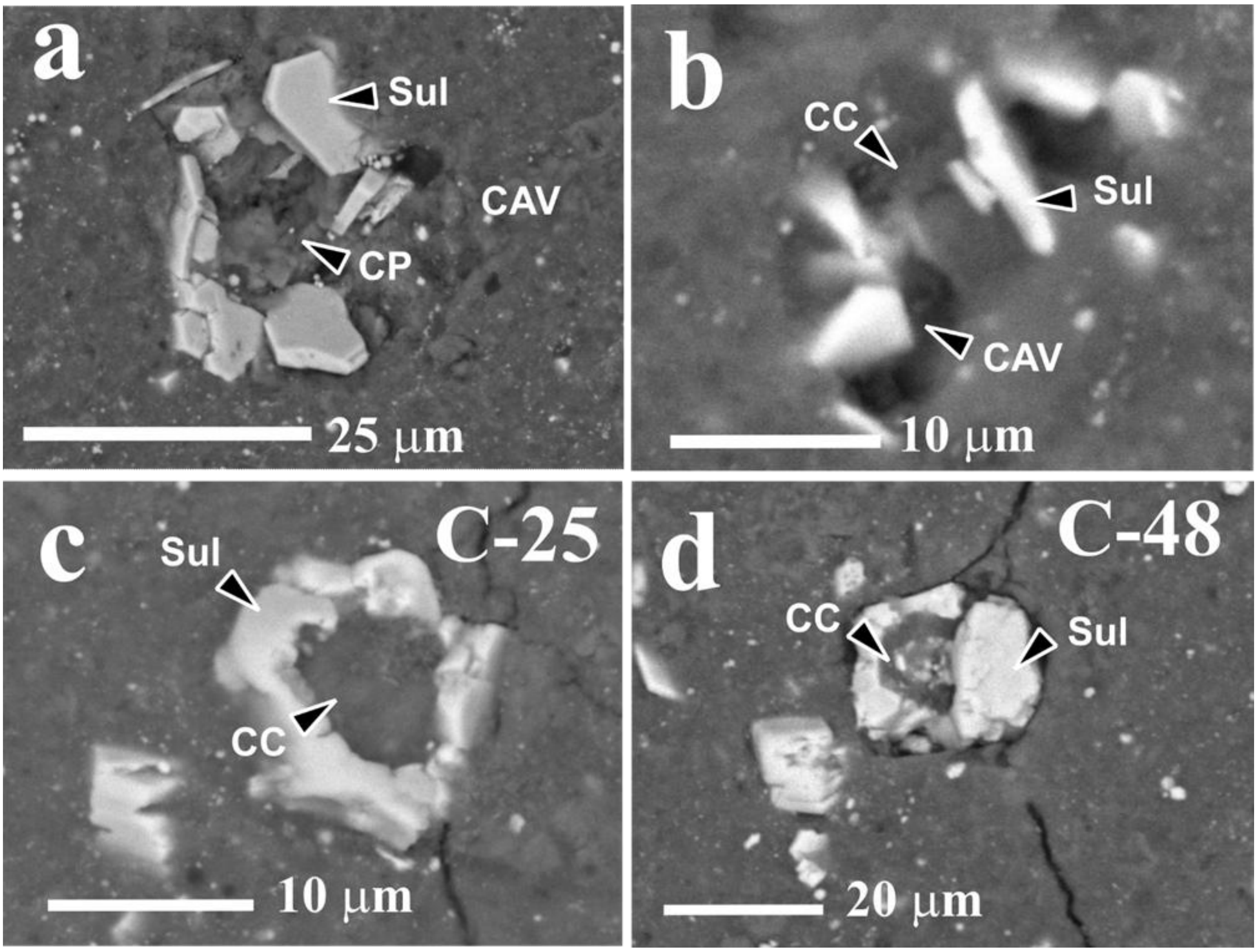

Supplementary Figure 5. Backscattered electron images of sulphide-rimmed objects in sample A0180. (a) A cavity with a partial rim of sulphide crystals and an interior filled with porous coarse phyllosilicate. These objects are larger than the majority of SSOs and are decorated with coarser crystals of sulphide. (b) A typical cavity in A0180 showing euhedral sulphides growing across a cavity

partially filled with porous coarse-phyllosilicates. Only a single cavity was observed (out of 37) with a rim of sulphide (shown in a). (c) A sulphide-rimmed object not interpreted as a microchondrule owing to its irregular faceted shape. (d) A sulphide-rimmed object not interpreted as a microchondrule owing to the facetted appearance of its core and external surface. Objects C-25 and C-48 were the only two observed during serial sectioning compared to 61 SSOs interpreted as microchondrules. Abbreviations: CC – cryptocrystalline core, CP – coarse-phyllosilicate, Sul – sulphide, CAV – cavity.

Supplementary Table 1. Compositions of SSO cores and matrix determined by electron microprobe in wt%. Low totals are the result of the volatile component. SSO C-02 experienced matrix overlap with surround iron-rich phases. Abbreviations: b.d – below detection.

	**Matrix**	**Matrix**	**C-03**	**C-03**	**C-03**	**C-04**	**C-04**	**C-04**	**C-02**	**C-02**
K2O	0.07	0.09	0.07	0.07	0.08	b.d	0.02	b.d	0.06	0.05
CaO	0.03	0.03	0.03	0.03	0.02	0.04	0.02	0.02	0.02	0.03
TiO2	0.06	0.06	0.09	0.08	0.08	0.07	0.08	0.09	0.10	0.07
P2O5	0.12	0.15	0.22	0.16	0.15	0.10	0.09	0.10	0.20	0.20
MnO	0.03	0.03	0.05	0.06	0.05	0.03	0.06	0.08	0.04	0.05
FeO	12.31	14.71	11.79	11.03	10.45	15.89	10.74	10.43	27.54	30.79
NiO	0.78	1.23	0.83	0.37	0.35	0.43	0.40	0.38	0.92	0.92
Cr2O3	0.48	0.48	1.06	1.07	1.10	0.76	0.99	1.05	0.89	0.79
Na2O	0.70	0.80	0.79	0.88	0.76	0.31	0.42	0.49	0.82	0.68
Al2O3	2.48	2.47	2.18	2.18	1.99	2.32	2.09	2.10	2.12	3.83
SiO2	40.67	37.76	39.82	40.94	38.96	33.97	36.27	36.83	24.83	20.92
MgO	23.13	21.13	21.98	21.93	20.35	19.31	19.70	20.29	15.01	12.51
Total	80.86	78.94	78.91	78.80	74.34	73.23	70.90	71.89	72.55	70.84

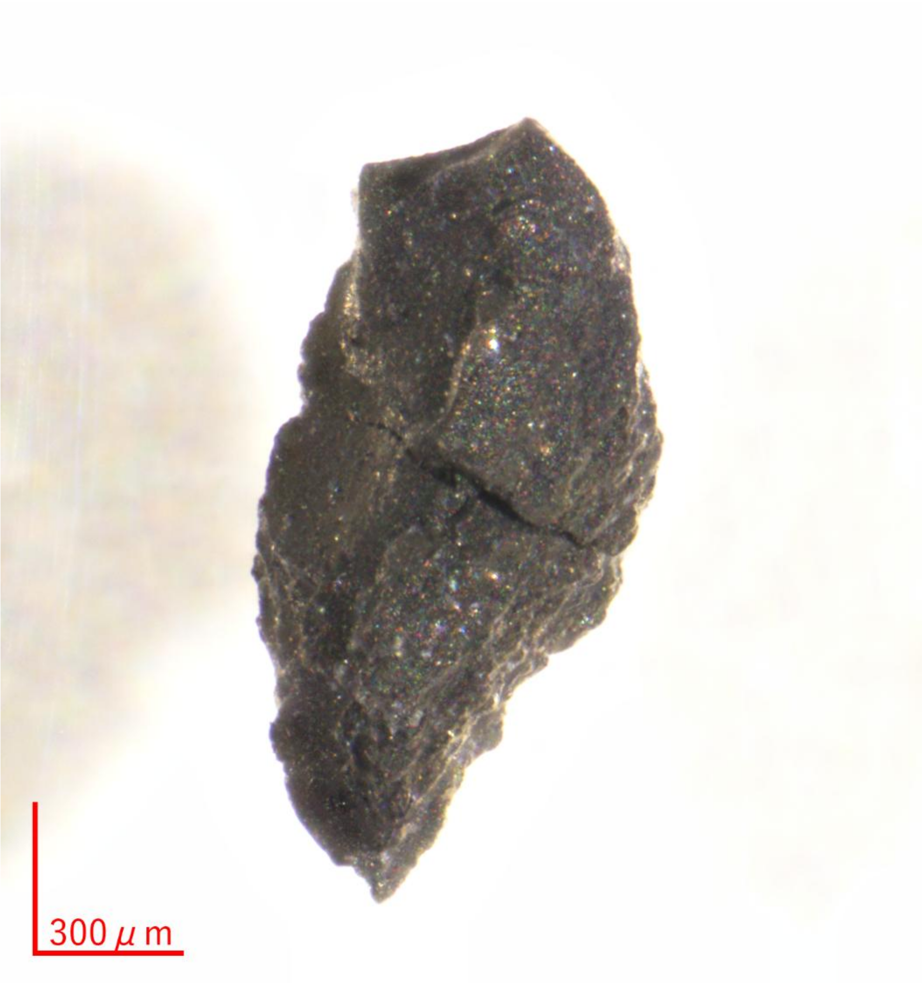

Supplementary Figure 6. An optical microscope image of particle A0180 showing facetted curved surfaces and a prominent fracture. The sample broke along the fracture. Sub-sample B is on the upper side of the fracture in this image. Credit: Ryugu Sample Database System, JAXA.

Supplementary Table 2. Results of reproducibility testing of the precision of manual segmentation of a large cavity. Volume, sphericity, elongation, flatness, and diameter measurements are shown in μm.

	Vol	Spher	Elong	Flat	Dmin	Dmax	Dmean
	7397.9	0.472	0.697	0.753	14.00	41.34	25.34
	6384.6	0.458	0.684	0.756	11.74	39.53	24.25
	6479.0	0.464	0.687	0.761	12.63	40.10	24.42
	6691.4	0.456	0.692	0.748	12.44	38.82	24.48
	6435.4	0.461	0.686	0.757	12.59	39.63	24.34
	6649.6	0.458	0.687	0.741	11.55	39.21	24.58
	6750.3	0.471	0.694	0.749	12.93	39.25	24.53
	6175.0	0.455	0.689	0.747	12.34	40.40	24.05
	6827.9	0.455	0.696	0.749	12.78	40.16	24.75
	7144.0	0.463	0.689	0.751	12.92	40.09	25.12
Mean	6693.5	0.461	0.690	0.751	12.59	39.85	24.59
STDEV	346.3	0.006	0.004	0.005	0.64	0.69	0.37
2σ	692.6	0.011	0.008	0.011	1.29	1.38	0.74
%	10.3	2.491	1.215	1.425	10.22	3.46	3.02
